# The Idiopathic Pulmonary Fibrosis Honeycomb Cyst Contains A Mucocilary Pseudostratified Epithelium

**DOI:** 10.1371/journal.pone.0058658

**Published:** 2013-03-20

**Authors:** Max A. Seibold, Russell W. Smith, Cydney Urbanek, Steve D. Groshong, Gregory P. Cosgrove, Kevin K. Brown, Marvin I. Schwarz, David A. Schwartz, Susan D. Reynolds

**Affiliations:** 1 Center for Genes, Environment and Health, National Jewish Health, Denver, Colorado, United States of America; 2 Department of Pediatrics, National Jewish Health, Denver, Colorado, United States of America; 3 Department of Medicine, University of Colorado School of Medicine, Aurora, Colorado, United States of America; University of Pittsburgh, United States of America

## Abstract

**Background:**

We previously identified a *MUC5B* gene promoter-variant that is a risk allele for sporadic and familial Idiopathic Pulmonary Fibrosis/Usual Interstitial Pneumonia (IPF/UIP). This allele was strongly associated with increased MUC5B gene expression in lung tissue from unaffected subjects. Despite the strong association of this airway epithelial marker with disease, little is known of mucin expressing structures or of airway involvement in IPF/UIP.

**Methods:**

Immunofluorescence was used to subtype mucus cells according to MUC5B and MUC5AC expression and to identify ciliated, basal, and alveolar type II (ATII) cells in tissue sections from control and IPF/UIP subjects. Staining patterns were quantified for distal airways (Control and IPF/UIP) and in honeycomb cysts (HC).

**Results:**

MUC5B-expressing cells (EC) were detected in the majority of control distal airways. MUC5AC-EC were identified in half of these airways and only in airways that contained MUC5B-EC. The frequency of MUC5B+ and MUC5AC+ distal airways was increased in IPF/UIP subjects. MUC5B-EC were the dominant mucus cell type in the HC epithelium. The distal airway epithelium from control and IPF/UIP subjects and HC was populated by basal and ciliated cells. Most honeycombing regions were distinct from ATII hyperplasic regions. ATII cells were undetectable in the overwhelming majority of HC.

**Conclusions:**

The distal airway contains a pseudostratified mucocilary epithelium that is defined by basal epithelial cells and mucus cells that express MUC5B predominantly. These data suggest that the HC is derived from the distal airway.

## Introduction

Alveolar scaring is a pathological hallmark of Idiopathic Pulmonary Fibrosis/Usual Interstitial Pneumonia (IPF/UIP) and this histological change parallels the disease-associated decrease in lung function. Thus, dysregulated alveolar epithelial-mesenchymal interactions have been investigated as a disease-initiating mechanism (reviewed in [1]). This hypothesis has drawn strength from the finding that fibroblastic foci (FF) are predictive of disease progression and from the spatial association of FF with alveolar type II (ATII) cell hyperplasia [2], [3].

However, histological and gene expression studies suggest that the airway is also involved in IPF/UIP. Bronchiolar lesions were identified in 14 of 16 IPF/UIP cases that involved bronchiolar hyperplasia with extension to the pleural surface [4]. Additionally, our analysis of gene expression in lung tissue demonstrated increased expression of airway epithelial cell-associated transcripts [5] including basal cell-specific keratins (K) (K5, K14), the airway secretory cell marker (PLUNC), and ciliated cell markers (FoxJ1 and various ciliary dynamins) in patients with IPF/UIP. Another study found basal cell dysplasia in bronchiolar-alveolar junctions, which extended to FF [6]. Histological analysis demonstrated that these basal cells expressed proteins involved in cell migration (laminin 5, fascin), extracellular matrix proteins (tenacin-C) and a wound-repair keratin profile (K6a, K13, K14) [6], [7]. Finally, we identified a common polymorphism in the mucin 5B (*MUC5B*) promoter that was associated with a markedly increased risk of developing both familial and sporadic IPF/UIP [8], [9]. This risk allele was associated with increased MUC5B expression in unaffected subjects and mRNA levels were 37-fold greater in those who were homozygous for the polymorphism [8].

Implication of MUC5B in IPF/UIP disease pathogenesis begs critical examination of the characteristics of the honeycomb cyst (HC), a mucin-containing structure in the IPF-UIP lung [10]. Some reports describe the HC epithelium as a cuboidal and alveolar-like [11], suggest a parenchymal derivation, and have focused attention on the ATII progenitor cell. Alternatively, a submucosal gland (SMG) origin was implicated by a mucin profile that was skewed toward MUC5B+ [12] and expression of NRG1α [12]. Finally, an airway origin for the HC is suggested by reports that the HC is mucus-filled and is lined by a pseudostratified and bronchiolar-like epithelium. Supporting this hypothesis, immunohistochemical analysis identified hyperplastic basal cells within the HC epithelium [4], [13]. Despite these similarities to the distal airway, the HC epithelium did not express CC10 [4], a normal terminal bronchiolar human airway marker [14], or the columnar epithelial transcription factor Nkx2.1 [12]. Collectively these data suggested that lung mucins and the airway epithelium are involved in the etiology of IPF/UIP but further studies were needed to reconcile inconsistencies in the literature.

In this study, we determined the gel-forming mucin (MUC5B and MUC5AC) expression profile in normal and diseased distal airways and contrasted this with expression in the HC. To better understand the origin of these mucus cells and the HC epithelium, we determined distal and HC epithelial sub-types using cell type-specific markers.

## Materials and Methods

### Ethics statement

The *National Jewish Health Institutional Review Board* is an independent committee designated by National Jewish Health. This committee is responsible for the following aspects of research involving human subjects: 1) review of proposed projects; 2) approval for initiating studies; and 3) periodic review of research. The primary purpose of such review is to assure the protection of the rifts and welfare of the human subjects. The National Jewish Health Institutional Review Board reviewed and approved the present study. A written informed consent was obtained for the original human work that produced the tissue samples.

### Study Population

De-identified formalin-fixed paraffin-embedded lower lobe lung tissue and data from 22 subjects with a diagnosis of IPF/UIP were obtained from the Lung Tissue Research Consortium (LTRC) which is supported by the National Heart, Lung, and Blood Institute (NHLBI) (http://www.ltrcpublic.com) (Table 1). Control formalin-fixed paraffin-embedded lung tissue from 19 subjects was obtained from the International Institute for the Advancement of Medicine (Edison, NJ). Control individuals exhibited no evidence of active infection or chest radiographic abnormalities, mechanical ventilation <48hr, PaO2/FiO_2_>200, and no past medical history of underlying lung disease or systemic disease that involved the lungs. These individuals suffered brain death and were consented for research at the time of transplant evaluation.

**Table 1:** Demographic Characteristics of Study Participants.

	Non-IPF/UIP Controls (n = 19)	IPF/UIP (n = 22)
Age, Mean Yr (IQR)	52.5 (44 – 58)	58.3 (56.3 – 62.8)
Sex, Males/Females	11/8	18/4
Smoking History*, Yes/No	11/8	15/2
Race/Ethnicity	19 - Caucasian/Non-Hispanic	21 - Caucasian/Non-Hispanic 1 –Hispanic

* Smoking history was not available for 5 IPF/UIP cases.

### Dual immunofluorescence (DIF) analysis

All staining was performed on 5 micron sections of lung tissue. Sections were cleared of paraffin by incubation at 60°C overnight and subsequent xylene washes. Sections were rehydrated using a graded ethanol series. Antigen retrieval was performed in 0.01 M Citric Acid pH 6.0 by microwaving the slides for 15 minutes at 1250 watts. Slides were blocked for 1 hour at room temperature using 5% BSA in PBS (blocking solution, BS). Primary antibodies were diluted in BS and applied to sections by overnight incubation at 4°C. The primary antibodies and dilutions were: MUC5AC (Neomarkers, MS145p1, 1∶500); MUC5B (a gracious gift from Dr. Dallas Swallow [15] 1∶200); MUC5B (Santa Cruz, SC-20119, 1∶200, used only when paired with anti-acetylated tubulin, ACT); ACT (Sigma, T6793, 1∶5000); Keratin 5 (Covance, PRB-160P, 1∶6000); pro-SPC (Seven Hills, WRAB-SPC, 1∶2000); Keratin 14 (Neomarkers, MS115p, 1∶500); Ki67 (DakoCytomation, TEC3, 1∶100). Alexa488 (green) and Alexa594 (red) labeled secondary antibodies (Life Technologies) were used to at a 1∶500 dilution in BS. Tissue sections that were stained with secondary antibody only were negative. Nuclei were stained with DAPI.

### Tissue Section Scoring

Distal and proximal airways and HC were identified as indicated in results using Aperio high-resolution H&E images. Staining patterns were scored by two independent observers as previously described [16]. Raw data by subject are presented in Fig S1.

### Statistical Analysis

Association between mucin positive distal airways (both MUC5AC and MUC5B) and IPF/UIP disease status was determined by a Fisher's Exact Test or Pearson's Chi-Square Test. Comparison of individual mucin staining categories was performed by test of proportions.

## Results

### Conducting airway regions

**Figure 1:**
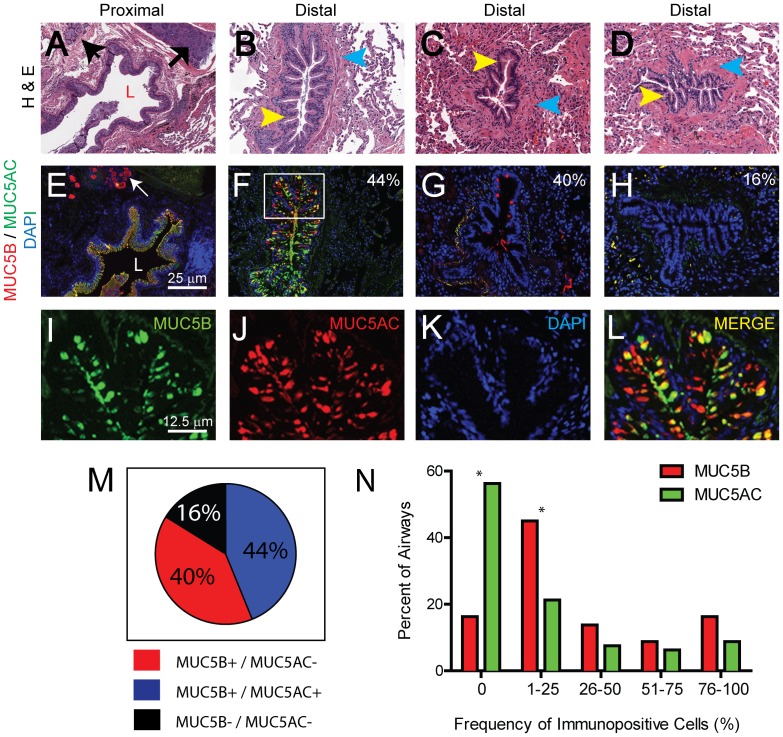
MUC5B is the dominant gel-forming mucin in the normal distal airway epithelium. A–D: Hematoxylin and eosin (H&E) staining of the normal conducting airway. A. Proximal airway; B–D. Distal airways. L, lumen; Arrow: cartilage; Arrowheads: black -submucosal gland, yellow - airway epithelium, blue - smooth muscle bands. Final magnification: 10× E–L: DIF analysis of MUC5B (red), MUC5AC (green) and nuclei (blue). E. Proximal airway. L: lumen; arrow: submucosal gland. F–H. Distal airways. Panels A & E; B & F; C & G; and D & H are adjacent sections. Representative images of airways expressing both MUC5B and MUC5AC (B, F); only MUC5B (C, G); neither MUC5B nor MUC5AC (D, H). Values in each panel indicate the frequency with which the pattern was detected. Final magnification, 10× I–L Single color images of the region indicated in panel F. Final magnification, 20×. M: Pie chart indicating the frequency of distal airways expressing MUC5B and/or MUC5AC. N: Frequency distribution of MUC5B (red) and MUC5AC (green) in distal airways. Asterisks represent significant p value (<0.05) obtained for test of proportions of that category.

The conducting airway can be divided into structurally and functionally distinct compartments [17]. Proximal airways were defined as those that were supported by cartilage and contained SMG (Fig 1A). The epithelium lining proximal airways was pseudostratified. DIF staining of proximal airways was used to test the specificity of two MUC5B antibodies (Fig S2). We confirmed that the proximal airway epithelium was populated by cells that expressed MUC5AC and co-expressed MUC5B and that the SMG epithelium was populated by mucus cells that expressed MUC5B and little MUC5AC (Fig 1E). Distal airways were defined as all airways that lacked cartilage and SMG (Fig 1B–D). These airways were surrounded by smooth muscle bands and contained deep invaginations.

### MUC5B is the dominant gel-forming mucin in the normal distal airway epithelium

Distal airways were identified in tissue sections from 84% (16 of 19) of control subjects. Mucus cell phenotype was evaluated in 80 distal airways (median per section = 5.5; Range = 2.0 – 7.0). DIF analysis of MUC5B and MUC5AC demonstrated that 44% (35 of 80) of distal airways were populated by both MUC5B-EC and MUC5AC-EC (Fig 1F, M). In these dual-positive airways, single-positive MUC5B or MUC5AC cells as well as numerous MUC5B/5AC double-positive cells were noted (Fig 1I-L). We found that 40% (32 of 80) of distal airways contained only MUC5B-EC (Fig 1G, M). Distal airways that expressed only MUC5AC were not detected. The remaining 16% (13 of 80) of distal airways were devoid of mucus cells (Fig 1H, M). In total, 84% of distal airways expressed MUC5B and 44% expressed MUC5AC. Distal airways that expressed MUC5AC were a subset of the MUC5B+distal airways.

To determine if the frequency of MUC5B-EC was different from that of MUC5AC-EC, distal airways were categorized according to the percent of epithelial cells that were positive for either mucin. The frequency of MUC5B+distal airways compared to MUC5AC+distal airways was higher in all positive staining categories, although this increase was significant only for the 1-25% category (Fig 1N).

### MUC5B and MUC5AC expression is increased in IPF/UIP distal airways

**Figure 2:**
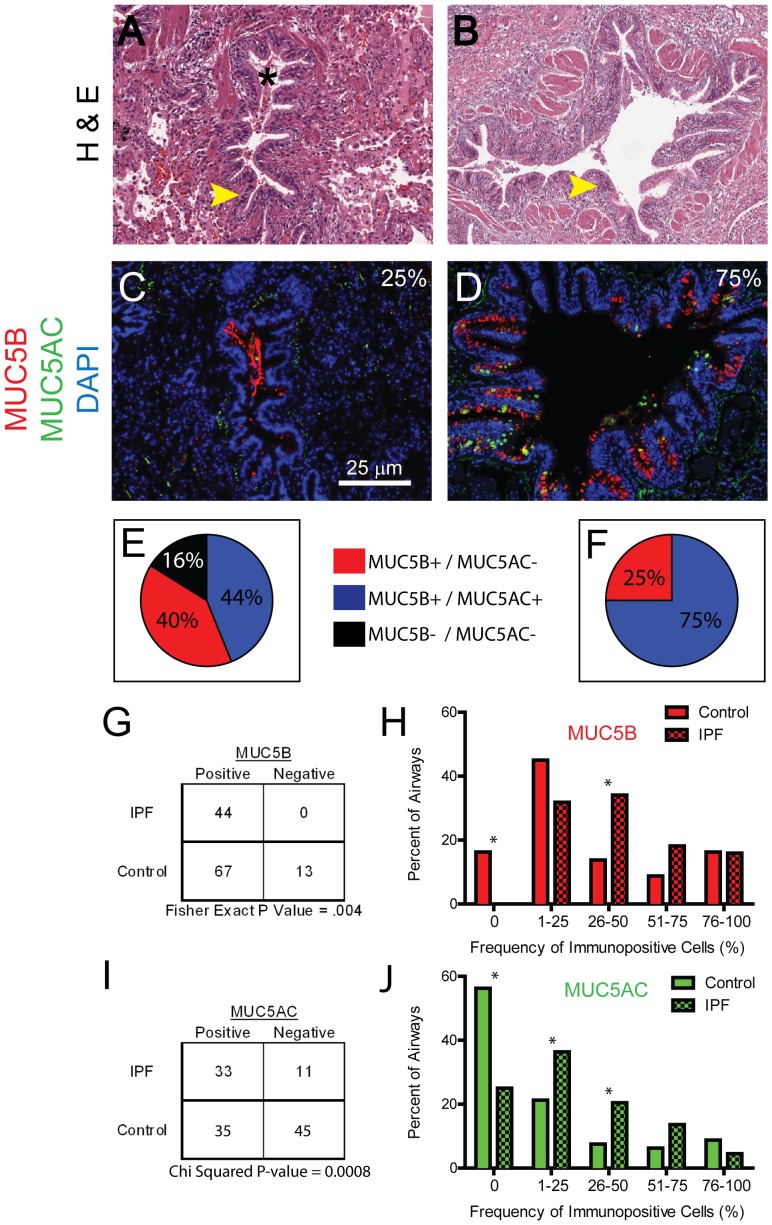
MUC5B and MUC5AC expression is increased in IPF/UIP distal airways. A–B: Hematoxylin and eosin (H&E) staining of the IPF/UIP distal airways. Asterisk-airway lumen; arrowhead - epithelium. Final magnification: 10× C-D: DIF analysis of MUC5B (red), MUC5AC (green) and nuclei (blue). Panels A & C; B & D are adjacent sections. Values in each panel indicate the frequency with which the pattern was detected. Final magnification,10× E-F: Pie charts indicating the frequency of distal airways expressing MUC5B and/or MUC5AC in normal subjects (E, represented from Fig 1N for comparison purposes) and IPF/UIP subjects (F). G: Frequency of MUC5B+distal airways in control and IPF/UIP patients. H. Frequency distribution of MUC5B-expressing cells in control (red, represented from Fig 1N for comparison purposes) and IPF/UIP (red-checked) subjects. I: Frequency of MUC5AC+distal airways in control and IPF/UIP patients. J. Frequency distribution of MUC5AC-expressing cells in control (green, represented from Fig 1N for comparison purposes) and IPF/UIP (green-checked) subjects. Asterisks in Fig 1H,J represent significant p value (<0.05) obtained for test of proportions of that category.

Microscopic honeycombing as well as structures with a distorted architecture and a simplified (less-cellular) epithelium complicated identification of distal airways in IPF/UIP lung sections. Thus, distal airways were identified by smooth muscle bands and a continuous epithelium that contained invaginations (Fig 2A–B). Distal airways were identified in tissue sections from 73% (16 of 22) IPF/UIP subjects (Fig 2A, B). Within these sections, 44 distal airways (median per section = 3, Range = 1.0 – 4.0) were scored for MUC5B and MUC5AC expression.

DIF analysis of MUC5B and MUC5AC demonstrated that all distal IPF/UIP airways were populated by mucus cells. In comparison to control (Fig 2E), all distal airways in IPF/UIP subjects contained MUC5B-EC (Fig 2C, D, F) and a subset of these airways (75%) also contained MUC5AC-EC (Fig 2D, F). This result was not driven by overweighting of one sample as averaging the MUC5B and MUC5AC expression data for airways within a single subject, showed that 81% of subjects expressed both MUC5B and MUC5AC in their airways. The frequency of MUC5B+ distal airways in IPF/UIP patients was significantly greater than that of control subjects (P = 0.004, Fig 2G). This difference was largely driven by an increase in the frequency of MUC5B+ IPF/UIP distal airways compared to controls in the 26–50% category (Fig 2H).

The frequency of MUC5AC+ distal airways in IPF/UIP patients was also significantly greater than that of control subjects (P = 0.0008, Fig 2I). This difference was driven by an increase in the frequency of MUC5AC+ IPF/UIP distal airways compared to controls in both the 1–25% and 26–50% categories (Fig 2H).

### MUC5B is the dominant gel-forming mucin in the HC

**Figure 3:**
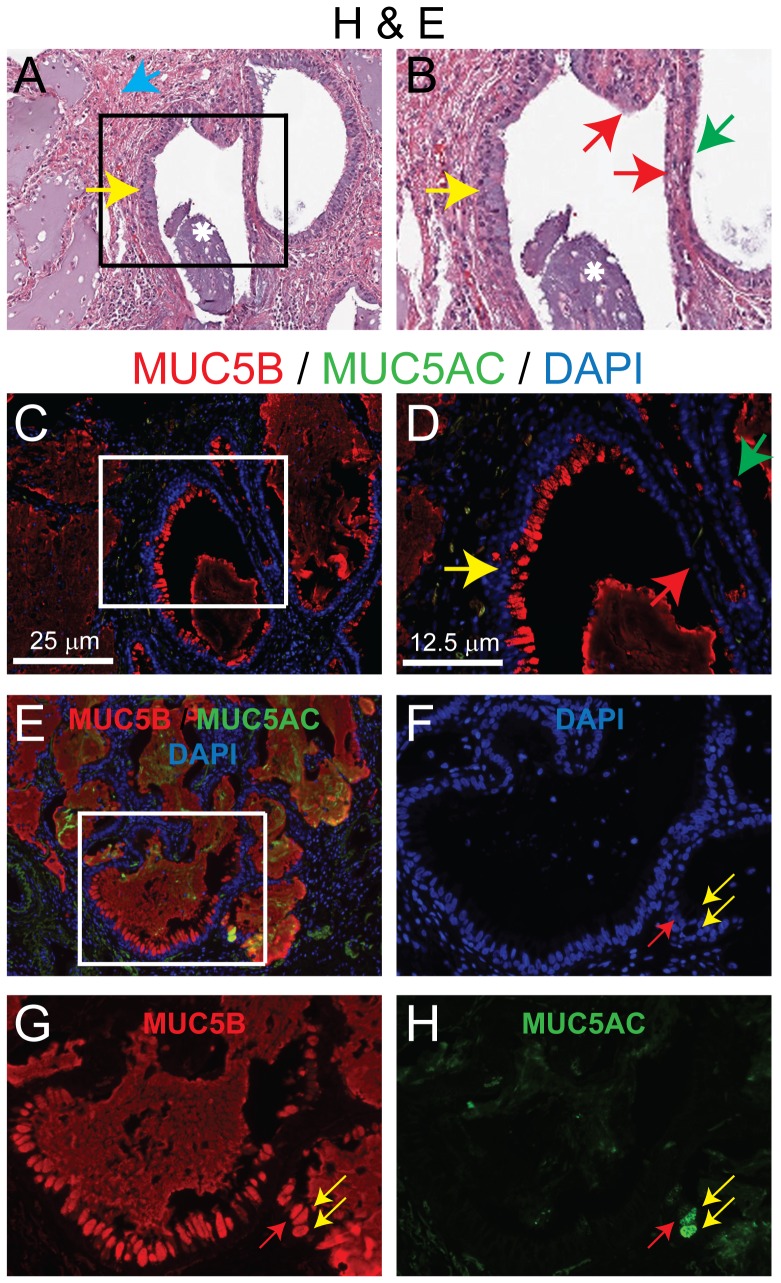
MUC5B is the dominant gel-forming mucin in the honeycombing cyst. A-B: Hematoxylin and eosin (H&E) staining of the honeycombing cyst. The region indicated in panel A (10×) is shown at higher magnification in B (20×). Arrows: yellow -pseudostratified epithelium, green - simple epithelium, red - disrupted epithelium, blue -fibrosis. Asterisk-mucus plug. C-H: DIF analysis of MUC5B (red), MUC5AC (green) and nuclei (blue). The region indicated in panels C (10×) is shown at higher magnification in panel D. D: Arrows: yellow -pseudostratified epithelium, green - simple epithelium, red - disrupted epithelium. The region indicated in panel E (10×) is shown at higher magnification in F-H (20×). Arrows: yellow MUC5B/MUC5AC dual-positive cells, red-MUC5B single-positive cell.

Areas of honeycombing were defined on H&E-stained sections as mucus-containing cysts that contained a less invaginated epithelium (Fig 3A, B) than that in the distal airways (Fig 2A, B). Most HC were lined by a distal airway-type epithelium that contained two subtypes, pseudostratified and simple (Fig 3A, B). Rare regions of the HC basement membrane were disrupted or denuded (Fig 3A, B). HC were associated with fibrotic tissue (Fig 3A, B) in contrast with the smooth muscle bands that surrounded normal distal airways (see Fig 2A, B). HC were identified in 68% (15 of the 22) of IPF/UIP subjects. Within these sections, 37 HC (median per section = 3, Range = 1.0 – 4.0) were scored.

DIF analysis of MUC5B and MUC5AC demonstrated that all HC were populated by MUC5B-ECs (Fig 3C, D) and that 35% of these harbored MUC5AC-EC (Fig 3E-H). All mucus cells were positioned within the luminal portion of the epithelium. Among the HC that contained MUC5B-EC and MUC5AC-EC, 92% of mucus cells **were** MUC5B+and 8% co-expressed MUC5AC.

### The HC contains a ciliated epithelium

**Figure 4:**
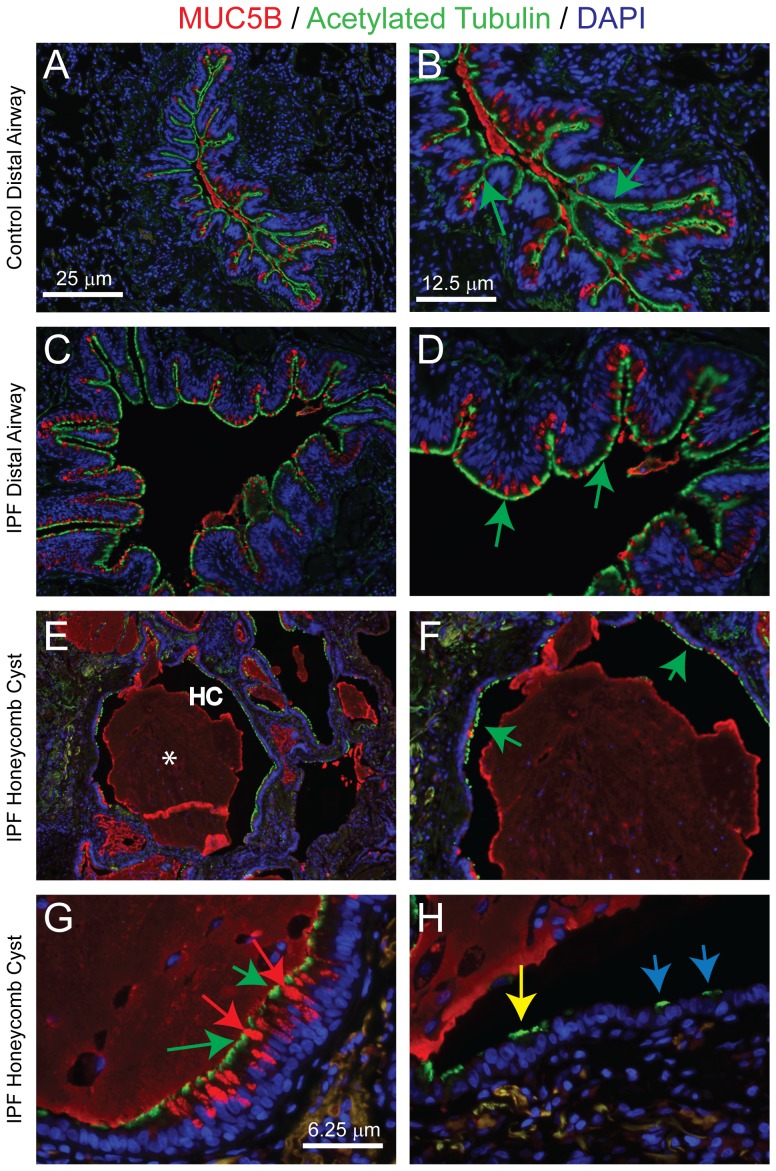
The honeycomb cyst contains a ciliated epithelium. A–F: DIF analysis of MUC5B (red), acetylated tubulin (ACT, green), and nuclei (blue). Control distal airway, A (10×) and B (20×). IPF/UIP distal airway, C (10×) and D (20×). Arrows: green - ciliated cells in the mucosecretory pseudostratified epithelium. Honeycomb cyst: E: (10×), F (20×), G-H (40×). Asterisks, mucus plug; HC, honeycomb cyst. Arrows: green - ciliated cells in the mucosecretory pseudostratified epithelium, red - MUC5B+cells in the mucosecretory pseudostratified epithelium, yellow - ciliated cells in the pseudostratified epithelium, blue - ciliated cells in the simple epithelium.

Similar to the epithelium that lines the distal airways, pseudostratified regions of the HC epithelium harbored mucus cells. Thus, we determined if the HC was populated by other airway-specific cell types. We first evaluated ciliated cells by immunostaining for acetylated-alpha tubulin (ACT). This marker detects the motile cilia that are diagnostic of ciliated airway epithelial cells. In agreement with the H&E staining (Fig 1B, 2B), ciliated cells were detected in the normal distal airways of control (Fig 4A, B) and IPF/UIP subjects (Fig 4C, D). Ciliated cells were uniformly distributed along the entire length of the airway epithelial surface but were not detected in the parenchyma.

Ciliated cells were also detected in the pseudostratified epithelium of the HC (Fig 4E, F) and most of these areas were uniformly ciliated. DIF analysis of ACT and MUC5B demonstrated that ciliated and mucus-producing cells were distinct cell types (Fig 4G). Areas of simple epithelium rarely contained ciliated cells (Fig 4H). Thus, the pseudostratified regions of the HC epithelium resembled, to a high degree, the epithelium of the normal distal airways.

### The HC contains basal cells

**Figure 5:**
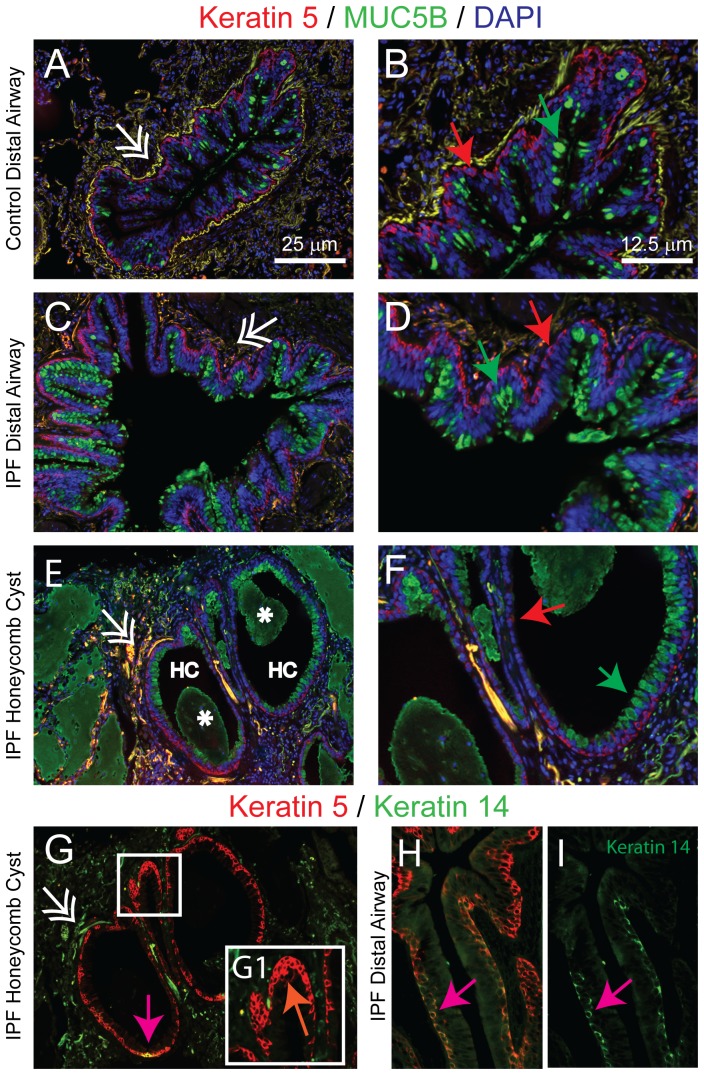
Basal cells populate the pseudostratified regions of the HC epithelium. A-F: DIF analysis of Keratin 5 (K5, red), MUC5B (green) and nuclei (blue). Control distal airway, A (10×) and B (20×). IPF/UIP distal airway, C (10×) and D (20×). Honeycomb cyst, E (10×) and F (20×). Asterisk, mucus plug; HC, honeycomb cyst. Double arrows: Auto-fluorescent red blood cells and elastin bands. Arrows: red - K5+cells; green - MUC5B+cells. G: DIF analysis of K5 and K14 in honeycomb cyst, G (10×). The region indicated in panel G is shown at higher magnification in G1 (20×). H, I: DIF analysis of K5 and K14 in IPF/UIP distal Airway (40×). Arrows: orange - dysplastic basal cells; pink - K5/K14 dual-positive cells.

Prior studies suggested the basal cell serves as the progenitor cell for both secretory and ciliated cells in normal human airways [18]. This cell is pyramidal in shape and is defined by expression of Keratin-5 (K5). As previously reported [17], K5+cells were detected in all distal airways of control and IPF/UIP subjects (Fig 5A-D) and were adjacent to the basement membrane (Fig 5B, D).

K5+cells were also identified in the HC epithelium (Fig 5E-H). In pseudostratified regions, the K5+cells exhibited a normal pyramidal shape and most were adjacent to the basement membrane. DIF analysis of K5 and MUC5B demonstrated that basal and mucus-producing cells were distinct cell types (Fig 5B, D, F). K5+cells were also identified in the simple epithelium of the HC (Fig 5F) and exhibited a patchy distribution. Although most HC-K5+cells were adjacent to the basement membrane rare areas of K5+cell hyperplasia were noted (Fig 5G). These cells were characterized by an abnormally high nuclear to cytoplasmic ratio (Fig 5G1).

To further investigate the properties of IPF/UIP basal cells, we stained for K14 and Ki67. K14 is a marker of basal cell mediated repair in the mouse [19] and is upregulated in abnormal regions of the human proximal airway [20]. Immunostaining detected rare K14+cells in 3 of 17 of IPF/UIP distal airways examined (Fig 5H, I). Most basal cells that were located in the pseudostratified HC epithelium were K14- (Fig 5G). However, rare K14+cells were detected within the simple epithelium in 10 of 11 HC (Fig 5G). DIF analysis of the proliferation marker Ki67 (not shown) demonstrated that the epithelial mitotic index was low in both the normal distal airway epithelium of IPF/UIP subjects and in the epithelium that lined the HC. Thus, the distal airway and HC epithelium contained normal basal cells in the pseudostratified regions and the patches of simple epithelium within HC contained reparative basal cells.

### Alveolar type II (ATII) cells are absent from the pseudostratified regions of the HC

**Figure 6:**
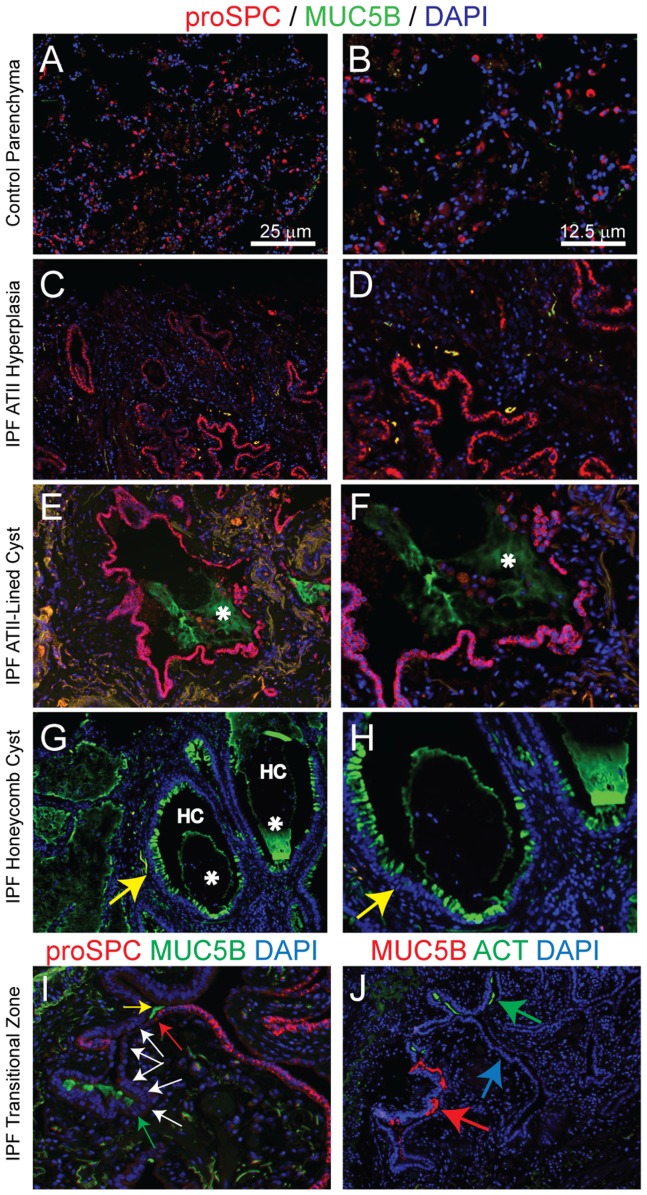
Alveolar type II (ATII) cells are absent from the pseudostratified regions of the HC. A-H: DIF analysis of pro-surfactant protein C (proSPC, red), MUC5B (green) and nuclei (blue). Control lung parenchyma, A (10×) and B (20×). IPF/UIP alveolar type II (ATII) cell hyperplasia, C (10×) and D (20×). IPF/UIP ATII-lined cyst, E (10×) and F (20×). Honeycomb cyst, G (10×) and H (20×). Asterisks, mucus plugs; HC, honeycomb cyst. I: IPF/UIP transitional zone. Arrows: yellow – pseudostratified epithelium. I-J IPF/UIP transitional zone. I: DIF analysis of pro-surfactant protein C (red), MUC5B (green), and nuclei (blue). Arrows: red - limit of the pro-SPC+simple epithelial region; yellow - luminal mucus; white - proSPC/MUC5B double-negative transition zone; green-MUC5B+pseudostratified region. J: DIF analysis of MUC5B (red), ACT (green), and nuclei (blue). Arrows, blue-simple epithelial region; red - MUC5B+cells, green-ACT+cells.

Prior work suggested that the HC might be derived from the alveolus and that HC epithelium expressed proSPC (reviewed in [11]), a protein characteristic of the ATII cell. However, there is disagreement on this point [4]. To address this issue, tissue sections from IPF/UIP patients were stained for proSPC. ProSPC+cells were identified in the normal parenchymal epithelium (Fig 6A, B). ATII cell hyperplasia was noted in non-honeycombing regions of most IPF/UIP sections (Fig 6C, D). Normal (Fig 6A, B) and hyperplastic ATII cells were negative for MUC5B (Fig 6C, D) and MUC5AC (not shown).

Interestingly, we observed examples in which proSPC+cells formed a simple cuboidal epithelium that lined a unique cystic structure. This epithelial lining was homogeneously proSPC+and did not express MUC5B (Fig 6E, F) or markers of basal or ciliated cells (not shown). These cysts were filled with MUC5B+mucus as well as nucleated non-mucus/proSPC- cells (Fig 6E, F).

Pro-SPC+cells were not detected in the pseudostratified regions of the HC (Fig 6G, H). However, rare proSPC+cells were detected in the simple epithelium within 11 of 33 HC (not shown). Within these HC, the frequency of proSPC+cells was less than 1% of epithelial cells.

In rare instances, the regions of ATII hyperplasia transitioned from a simple proSPC+epithelium to a pseudostratified epithelium that contained MUC5B+cells (Fig 6I) but no detectable keratin 5+basal cells (not shown). Interestingly, the simple and pseudostratified epithelia were often separated by a region of simple epithelium that was devoid of both proSPC-EC and MUC5B-EC. In some instances, the simple epithelium transitioned to regions that contained either ciliated cells or MUC5B-EC (Fig 6J). The scarcity of these transitional zones prevented quantification.

## Discussion

We recently discovered a genetic variant in the MUC5B gene promoter that was strongly associated with the development of IPF/UIP [8]. Further, we showed that this variant was associated with increased MUC5B lung gene expression in unaffected-control patients [8]. These results raised the possibility that mucus secretions and/or the compartment that produces these secretions play a role in the pathogenesis of IPF/UIP. To begin to address this hypothesis we compared the mucin profile and cellular phenotypes in control distal airways, IPF/UIP distal airways, and HC.

We showed that the majority of control distal airways contained MUC5B-EC and that a subset of these airways harbored MUC5AC-EC. Normal distal airways did not express MUC5AC in the absence of MUC5B. This distal mucin pattern was in contrast with the accepted expression pattern in proximal airways (i.e. trachea and bronchi) where MUC5AC is the dominant mucin and fewer MUC5B-EC are detected. Collectively, our data demonstrate that MUC5B is preferentially expressed in the normal distal airway epithelium and that this pattern distinguishes the distal and proximal airways.

We found that MUC5B+distal airways were more frequent in the IPF/UIP lung relative to control subjects (Fig 2). A parallel increase in the frequency of MUC5B-EC suggested that this aspect of IPF/UIP disease pathology involved two processes: conversion of MUC5B- distal airways to MUC5B+airways and increased frequency of MUC5B-EC in distal airways that were already positive. The molecular mechanism that regulates this process may involve the MUC5B promoter polymorphism but is likely to also include complex gene by environment interactions that cannot be evaluated using end-stage histological sections.

Previous investigators indicated that the HC was derived from the bronchiolar while others indicated that it was derived from the alveolar epithelium (reviewed in [11]). We observed numerous areas of ATII hyperplasia some of which formed mucus-filled cystic structures. This observation suggested that ATII hyperplasia could evolve into cystic structures. We also showed that these ATII cysts were lined by a simple epithelium composed of mucin-negative, proSPC+ATII cells. Finally, our study demonstrated that the pseudostratified HC epithelium was devoid of proSPC+cells. These data lead us to hypothesize that the IPF/UIP lung contains subclasses of cysts, ATII cysts and HC, and that the ATII cyst was derived from the alveolar epithelium.

In contrast with the ATII cyst, we showed that a pseudostratified epithelium composed of mucus, ciliated, and basal cells was present in the HC. We found that MUC5B-EC were the dominant (95%) mucin-expressing cell type in the HC. This mucin profile aligns closely with the mucin profile of “bronchiolar lesions” that was reported recently [12]. However, our characterization of distal airways revealed that the HC mucin profile was more similar to that of the normal distal airway than the proximal airway (Fig 1) and more similar to the IPF/UIP distal airway than to the normal distal airway. In contrast with the hypotheses that the HC originates in the SMG [12] or the alveolar epithelium (reviewed in [1]), our analysis suggests that the HC is derived from the distal airway. The absence of mucus cells in ATII cysts suggested that the mucus plug originated in the distal airways. However, we acknowledge that expression of airway markers by ATII cells, as demonstrated in rodents [21], [22], or differentiation of a human multilineage stem cell could also explain these results [23].

We previously reported that basal cell-associated keratins (K4, K5, and K15), an airway-specific secretory protein (Plunc), and ciliary proteins (FoxJ1, and various dynamins) were over-expressed in the IPF/UIP lung relative to control [5]. We now show that the HC mucus cell profile is similar to the distal airway, that pseudostratified regions of the HC epithelium are ciliated (Fig 5), and that these regions are lined by basal cells (Fig 6). While histological analysis of advanced disease tissue is a limitation of this study, our data lead us to speculate about the genesis of the HC.

When considering the origin of the HC, first question that comes to mind is, “are the HC simply damaged airways (Process A) or do they result from bronchiolarization of the airspace (Process B)? Process A may be the consequence of parenchymal injury and consequent fibrosis, loss of the intervening parenchyma, and contraction that causes distal airways to coalesce. In this scenario, honeycombing would be a consequence of traction bronchiectasis [24].

Alternatively, Process B may be a consequence of an airway progenitor cell's response to the aberrant injury and repair that is known to occur in IPF/UIP lungs [25]. Previous studies reported that IPF/UIP basal cells exhibited a migratory phenotype [6]. Accordingly, we suggest that disease-related injury/repair may stimulate basal cell migration into the airspace. Once there, the basal progenitor cells may proliferate and differentiate. Normal basal cell differentiation is known to result in generation of a pseudostratified mucociliary epithelium similar to that identified in the HC [18], [26]. However, the IPF/UIP MUC5B risk-allele may cause accumulation of mucus with a MUC5B-skewed mucin profile. Occlusion of the upstream airway would then cause further damage to the downstream lung parenchyma. This “distal airway outgrowth model” accommodates our identification of an IPF/UIP risk variant in the airway gene *MUC5B*, gene expression data that implicate the distal airways in the pathogenesis of IPF/UIP, previously published radiological evidence of connectivity between the airway and the HC, histological analyses of progressive heterogeneous parenchymal destruction, and the present DIF identification of airway cell types in the HC.

## Supporting Information

Figure S1 **MUC5B and MUC5AC expression in control and IPF/UIP distal airways.** Distal airways of control and IPF/UIP subjects were categorized according to the frequency of MUC5B or MUC5AC-expressing cells. Five categories were established: those in which immunopositive cells were 0%, 1–25%; 26–50%; 51–75%; or 76–100%, of all cells. Data for all subjects (each symbol represents a single subject within the control or IPF/UIP plots) are presented for: A. control - MUC5B; B. control - MUC5AC; C. IPF/UIP - MUC5B; and D. IPF/UIP - MUC5AC. Rubric: the control subject represented by the red circle had 3 distal airways in the MUC5B 0% category, 3 distal airways in the 1–25% category and no distal airways in the 26–50%, 51–75%, or 76–100% categories. In contrast, the control subject represented by the yellow square had no airways in the MUC5B 0% and 1–25% categories, 1 airway in the 26–50% category, 2 distal airways in the 51–75% category, and 3 airways in the 76–100% category.
(TIF)

Figure S2 **MUC5B antibody validation.** A–F: DIF analysis of the Swallow [15] (red) and Santa Cruz (green) MUC5B antibodies. All images are of the same region. A–C are single-color images at (40X), D–E are dual-color merged images, F Triple-color merged image. M, mucus. Arrows: Examples of cells detected by both antibodies.
(TIF)
