# Supplementary figures and images for: The Idiopathic Pulmonary Fibrosis Honeycomb Cyst Contains A Mucocilary Pseudostratified Epithelium
Source: PLoS One. 2013 Mar 20;8(3):e58658. doi: 10.1371/journal.pone.0058658 (PMC3603941; doi:10.1371/journal.pone.0058658)

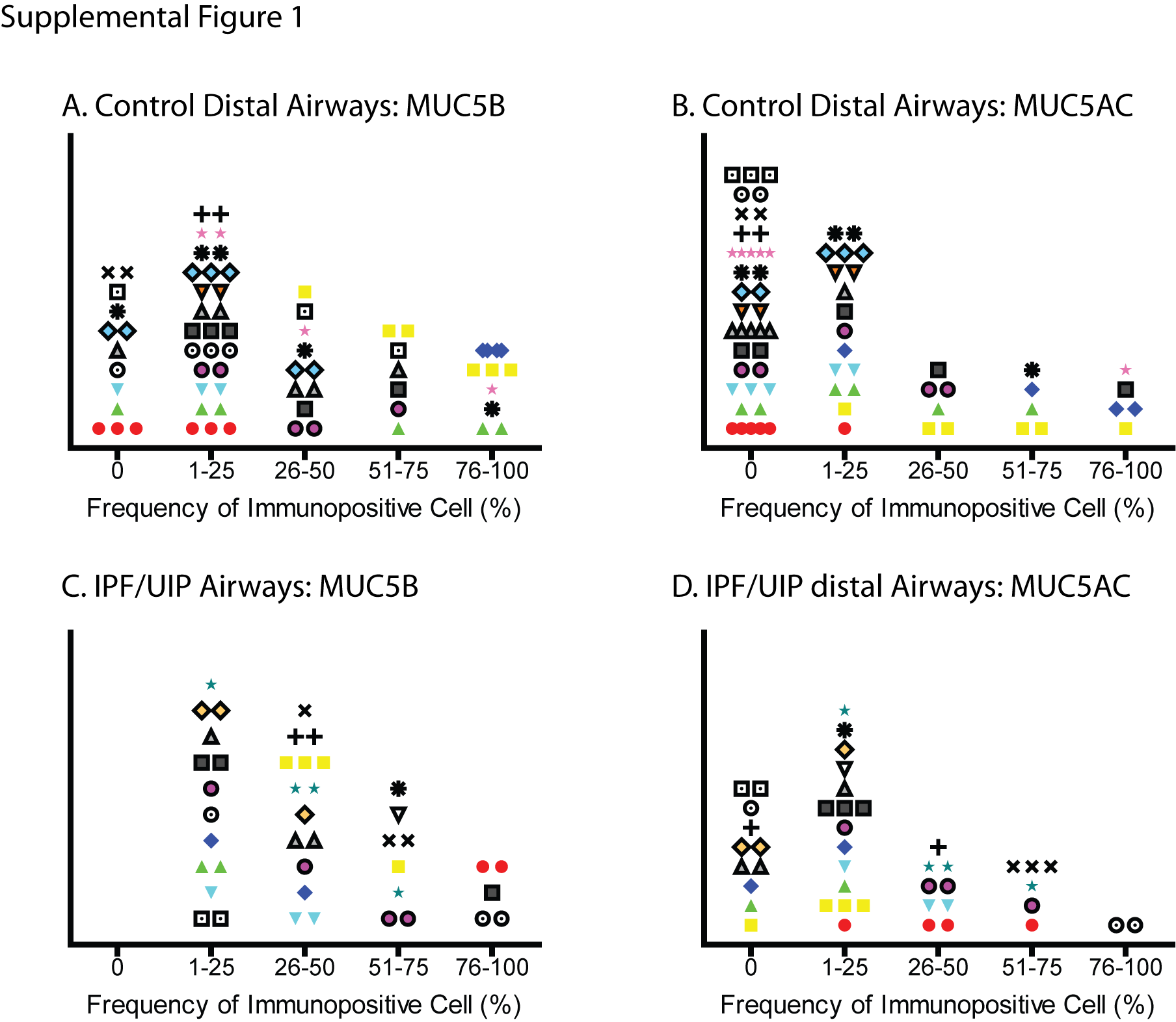

Supplement: Figure S1 — MUC5B and MUC5AC expression in control and IPF/UIP distal airways. Distal airways of control and IPF/UIP subjects were categorized according to the frequency of MUC5B or MUC5AC-expressing cells. Five categories were established: those in which immunopositive cells were 0%, 1–25%; 26–50%; 51–75%; or 76–100%, of all cells. Data for all subjects (each symbol represents a single subject within the control or IPF/UIP plots) are presented for: A. control - MUC5B; B. control - MUC5AC; C. IPF/UIP - MUC5B; and D. IPF/UIP - MUC5AC. Rubric: the control subject represented by the red circle had 3 distal airways in the MUC5B 0% category, 3 distal airways in the 1–25% category and no distal airways in the 26–50%, 51–75%, or 76–100% categories. In contrast, the control subject represented by the yellow square had no airways in the MUC5B 0% and 1–25% categories, 1 airway in the 26–50% category, 2 distal airways in the 51–75% category, and 3 airways in the 76–100% category. (TIF) [file pone.0058658.s001.tif]

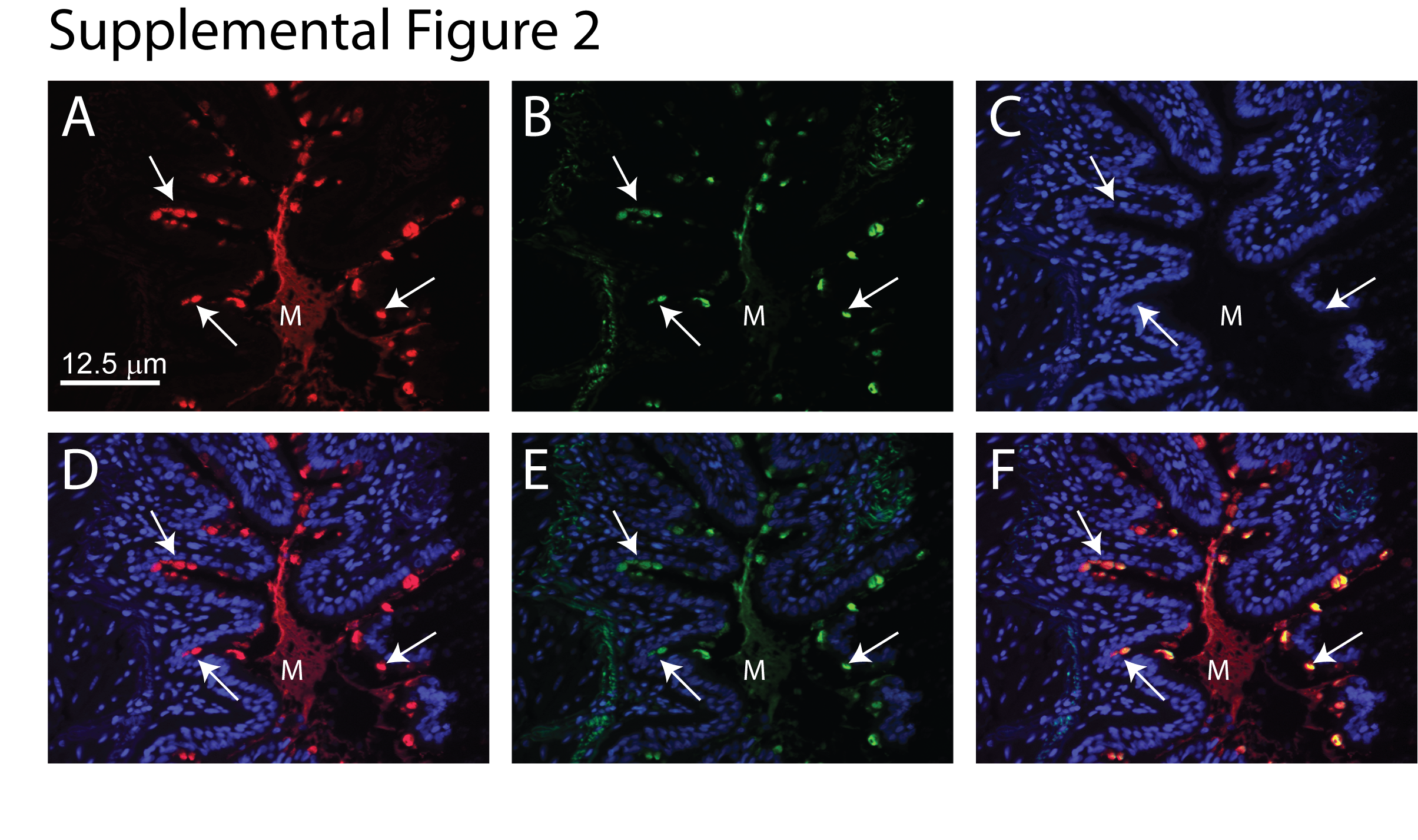

Supplement: Figure S2 — MUC5B antibody validation. A–F: DIF analysis of the Swallow [15] (red) and Santa Cruz (green) MUC5B antibodies. All images are of the same region. A–C are single-color images at (40X), D–E are dual-color merged images, F Triple-color merged image. M, mucus. Arrows: Examples of cells detected by both antibodies. (TIF) [file pone.0058658.s002.tif]
